# Isolation and characterization of Avs-1, a bacteriophage effective against the aquaculture pathogen *Aeromonas veronii*

**DOI:** 10.1128/aem.02334-25

**Published:** 2026-05-13

**Authors:** Chen Li, Jiayi Liu, Xinran Long, Linger Qian, Jiajie Xie, Chaoqun Luo, Xiaorong Yang, Yanming He, Yushan Zhang, Hui Cai, Jiayi Wang, Fan Yang, Ming Jang, Hongrong Liu, Shengbiao Hu

**Affiliations:** 1 Hunan Provincial Key Laboratory of Microbial Molecular Biology, College of Life Science, Hunan Normal University 12568, Changsha, China; 2 Institute of Interdisciplinary Studies, Key Laboratory for Matter Microstructure and Function of Hunan Province, Key Laboratory of Low-dimensional Quantum Structures and Quantum Control, School of Physics and Electronics, Hunan Normal University 12568, Changsha, China; University of Nebraska-Lincoln, Lincoln, Nebraska, USA

**Keywords:** *Aeromonas veronii*, bacteriophage, cryo-electron microscopy, phage resistance, endolysin

## Abstract

*Aeromonas veronii* is a representative pathogen resulting in significant economic losses in aquaculture. As a potential antibacterial agent, bacteriophage has attracted wide attention. Herein, a virulent phage named Avs-1 that infects *Aeromonas veronii* was isolated from sewage. Avs-1 demonstrated an optimal multiplicity of infection (MOI) of 0.1, with a 20-min latent period and a 130-min burst period. Cryo-electron microscopy structural analysis revealed that Avs-1 possesses an icosahedral capsid composed of 415 copies of the major capsid protein arranged in a *T* = 7 lattice, along with a long non-contractile tail typical of the *Siphoviridae* family. Phage resistance in strain AV0110 was linked to a frameshift mutation in the *manB* gene encoding phosphomannomutase (PMM). The genome of phage Avs-1 consists of 44,364 bp double-stranded circular DNA, in which the endolysin (Lys40) has a modular structure with a N-acetylmuramidase domain at N-terminus and a peptidoglycan (PG)-binding domain at C-terminus. Notably, endogenous expression of Lys40 could greatly inhibit the growth of *E. coli* but could not lyse them. Avs-1 exhibited significant antibacterial effect *in vitro* and increased tilapia survival rate by 50% when used *in vivo*. This study provides a theoretical basis for further research on the interaction between pathogens and phages, and the prevention and treatment of *Aeromonas veronii* and highlights the potential of Avs-1 as a targeted biocontrol agent against *Aeromonas veronii* infections in aquaculture.

**IMPORTANCE:**

In this study, we present an eco-friendly approach to address a critical issue in aquaculture: *Aeromonas veronii* infections, which cause significant economic losses. We turned to nature’s bacterial predators—bacteriophages and viruses that specifically infect and kill bacteria. From sewage samples, we isolated a novel phage named Avs-1, which effectively targets *A. veronii*. Genomic and structural analyses were conducted to elucidate its mechanisms. Notably, we also identified a key gene that enabled bacteria to resist phage adsorption, which provides critical insights for future phage applications. Most importantly, in our laboratory tests, the Avs-1 bacteriophage demonstrated potent antibacterial activity and increased the survival rate of tilapia infected with *A. veronii* by 50%. The potential of Avs-1 bacteriophage as a targeted, environmentally friendly “biological pesticide” for controlling infections in aquaculture offers a promising alternative to conventional antibiotics.

## INTRODUCTION

The intensification of production has led to a deterioration of the aquaculture water environment, resulting in frequent occurrences of bacterial diseases (1, 2). Among these, Aeromonas is the most prevalent pathogen in freshwater culture, encompassing various species, such as *Aeromonas hydrophila* (*A. hydrophila*), *Aeromonas veronii* (*A. veronii*), and *Aeromonas salmonicida* (*A. salmonicida*) (3). *A. veronii*, a Gram-negative pathogen that co-infects humans, animals, and aquatic organisms, widely exists in natural environments, such as freshwater lakes and soil, leading to sepsis, ulcers, intestinal inflammation, and other diseases and causing serious economic losses to aquaculture industries around the world and posing a threat to public health security (4–7). Antibiotics are commonly used to control diseases caused by bacteria such as *Aeromonas* and are effective (8). However, the misuse of antibiotics contributes to the rise of multidrug-resistant bacteria, presenting a significant challenge in the prevention and treatment of pathogenic infections (9). Various studies have demonstrated that *A. veronii* exhibits resistance to multiple antibiotics (10, 11). Consequently, there is a pressing need to explore alternative therapies for combating diseases caused by *A. veronii* infections.

Bacteriophages (or phages) are viruses that infect and multiply within living bacterial cells, utilizing the bacterial transcription and translation machinery to complete their replication cycle (12). They are commonly found in various environments such as soil, water, food, and the gastrointestinal tract (13). Due to their harmlessness to the host microbiota, host specificity, rapid reproduction, and cost-effectiveness, phages show great potential as an effective alternative for controlling bacterial diseases (14). Remarkably, several studies have demonstrated the potential of phage therapy to significantly reduce or even prevent mortality in fish infected with *Aeromonas*, highlighting its efficacy (15–17). Although extensive research on their applications has been carried out, research on the structure of bacteriophages remains limited. Cryo-electron microscopy (Cryo-EM) has played a significant role in resolving the structure of bacteriophages, providing high-resolution atomic-level structural information and revealing the assembly mechanism of bacteriophages, the infection process, and their interaction with the host (18–21). Similar to bacterial resistance to antibiotics, bacterial resistance to phages is also occurring (22, 23). Bacteria continue to evolve in the arms race with phages, evading phage targets, such as ceasing expression of receptor genes due to mutations or expressing structurally defective proteins to escape phage adsorption (24).

Typically, double-stranded DNA phages employ a two-component lysis system consisting of holin and endolysin (25). In this system, holin perforates the bacterial inner membrane, allowing endolysin to translocate into the periplasmic space, where endolysin subsequently degrades the host bacterial cell wall (26). Holins are a class of membrane proteins characterized by small molecular weights and the presence of at least one predicted transmembrane domain (TMD), which determines the phage infection cycle (27). Endolysin, also referred to as lyase, is a peptidoglycan hydrolase encoded by double-stranded DNA phages (28). Its primary function is to degrade the peptidoglycan layer of the bacterial cell wall, facilitating the release of viral particles (29). The structure of endolysin depends on the host of the phage (30). Typically, the structure of endolysins from phages that infect Gram-positive bacteria is modular, consisting of enzymatically active domains (EADs) and cell wall-binding domains (CBDs) (31). In contrast, in phages that infect Gram-negative bacteria, endolysins consist only of an EAD (32).

In this study, we isolated a virulent phage (designated as Avs-1) from sewage using *A. veronii* AV1212 as the host and an Avs-1-resistant strain, *A. veronii* AV0110. The frameshift mutation in the *manB* gene, encoding phosphomannomutase (PMM) in AV0110, was identified by analyzing the genome sequence of two strains of AV1212 and AV0110 and was shown to be the cause of AV0110’s resistance to Avs-1. We analyzed the optimal multiplicity of infection (MOI), growth characteristics, and Cryo-EM structure of Avs-1. A potential endolysin (designated Lys40) was identified in the Avs-1 genome, which can significantly inhibit the growth of *Escherichia coli* (*E. coli*) but cannot lyse it. This study provides comprehensive structural and genetic insights into phage Avs-1, laying a foundation for its future application in controlling *A. veronii* infections in aquaculture.

## RESULTS

### Isolation and characterization analysis of Avs-1

**Fig 1:**
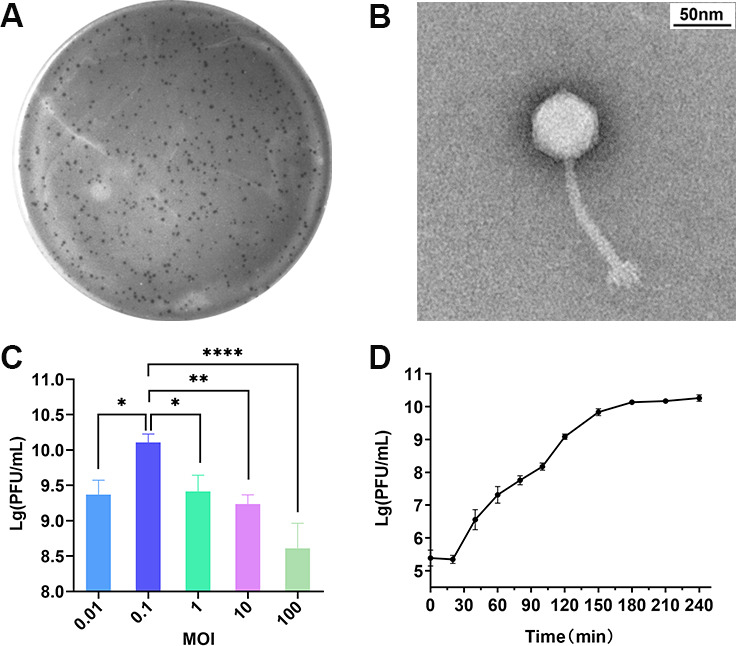
Isolation and morphology of Avs-1. (**A**) Plaques formed on *A. veronii* AV1212 agar plate. (**B**) Transmission electron micrographs reveal the morphology of phages. Scale bar = 50 nm. (**C**) Determination of optimal multiplicity of infection (MOI) of phage of Avs-1. (**D**) One-step growth curve determination of Avs-1. The dates were expressed as mean values ±SD and were analyzed using two-way ANOVA. * indicates *P* < 0.05; ** indicates *P* < 0.01; and **** indicates *P* < 0.0001.

Bacteriophage Avs-1 was isolated from sewage using *A. veronii* AV1212, preserved in our laboratory, as the indicator bacterium, and formed clear phage plaques on the *A. veronii* AV1212 lawn after purifications (Fig. 1A). Transmission electron microscopy (TEM) revealed that Avs-1 has an icosahedral head and a long non-contractile tail (Fig. 1B). Based on these morphological characteristics and according to the latest International Committee on Taxonomy of Viruses (ICTV) classification (33), phage Avs-1 was classified into the *Siphoviridae* family. Avs-1 could only infect host bacterium *A. veronii* AV1212 and not other tested *Aeromonas* species and related genera (Table 1), which reduces damage to other probiotics and increases its potential as a biological agent. The phage titer reached the highest as 1.31 × 10 ^10^ PFU/mL when the MOI was 0.1, and the optimal MOI of bacteriophage Avs-1 was 0. 1 (Fig. 1C). At this MOI, we analyzed the one-step growth curve of phage Avs-1 to determine its growth characteristics. The results showed that the latent period of Avs-1 was 20 min, followed by a burst period of 160 min, reaching a burst size of approximately 87 plaque-forming units (PFU)/cell (Fig. 1D).

**TABLE 1:** Detection of host range of phage Avs-1

Bacterial strain	Spot of Avs-1^*a*^	Strain resource
*Aeromonas veronii* AV1212	+	Lab collections
*Aeromonas allosaccharophila*	−	Lab collections
*Aeromonas salmonicida*	−	Lab collections
*Aeromonas jandaei*	−	Lab collections
*Aeromonas hydrophila*	−	Lab collections
*Aeromonas sobria*	−	Lab collections
*Edwardsiella tarda*	−	Lab collections
*Citrobacter freundii*	−	Lab collections
*Plesiomonas shigelloides*	−	Lab collections
*Escherichia coli* BL21(DE3)	−	Lab collections

^*a*^ +, plaques were observed; −, plaques were not observed.

### Overall structure of Avs-1 phage

**Fig 2:**
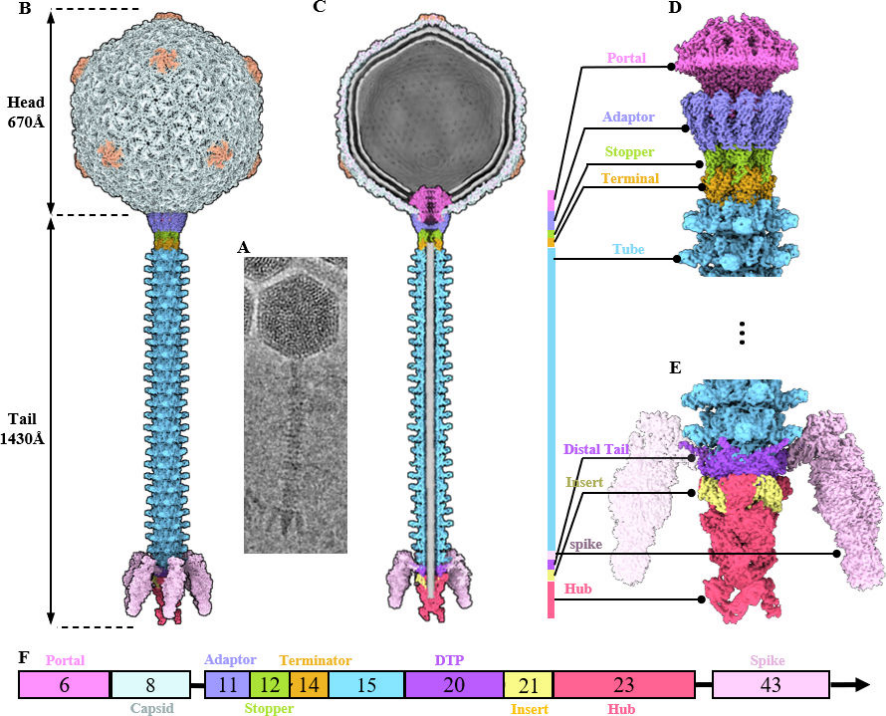
Overall structure of siphophage Avs-1. (**A**) Zoomed-in view of a particle image of Avs-1. (**B, C**) Side (**B**) and cut-open (**C**) views of the asymmetric structure of Avs-1. (**D, E**) Side view of the density maps of the connector (portal gp6, adaptor gp11, stopper gp12) and the tail tube (tail terminator gp14, and tail tube gp15). (**E**) Side view of the density maps of the tail tip (distal tail gp20, insert gp21, hub gp23, and spike gp43). (**F**) Scheme of Avs-1 genome segment encoding structural genes. The color code is applied to panels B to E.

The mature Avs-1 phage, which was used for the data collection of cryo-EM, was isolated and purified from sewage after amplification in *A. veronii* AV1212 (Fig. 2A). A total of 44,943 mature particles were extracted from 3,068 micrographs. Using cryoSPARC software (34), we reconstructed the head structure of Avs-1 at a resolution of 2.98 Å by imposing icosahedral symmetry (Fig. 2B and C). After 2D classification to select particles containing the head and the connector, we resolved the connector-tail complex at 3.1 Å resolution (Fig. 2D). Subsequently, we focused on the regions of the tail tube, tail tip, and tail spike and resolved their high-resolution structures to 3.26 Å, 3.62 Å, and 3.58 Å, respectively (Fig. 2E). The high-resolution maps allowed us to build the atomic models for the major capsid protein (MCP) gp8, portal protein gp6, adaptor protein gp11, stopper protein gp12, terminal protein gp14, tail tube protein (TTP) gp15, distal tail protein (DTP) gp20, hub protein gp23, insertion protein gp21, spike protein gp43, and the C-terminus of tape measure protein (TMP) gp19. However, the N-terminus of TMP could not be resolved, likely due to its flexibility.

The Avs-1 head, with a diameter of 670 Å, is composed of 415 copies of MCP gp8, each of which adopts a canonical HK97-like fold (35). All the 415 MCP monomers are organized into capsomers, including 11 pentons and 60 hexons, which are ultimately assembled into an icosahedral shell with a triangulation (T) number of 7. The connector, which occupies a unique fivefold vertex of the head, serves as a channel for DNA package and delivery (Fig. S1A). Structurally, the connector is constituted by a dodecameric portal protein gp6, a dodecameric adaptor protein gp11, and a hexameric stopper protein gp12 (Fig. S1). These proteins display structural conservation to those of siphophages lambda, T5, and T1 (36–38).

The Avs-1 tail tube is constituted of a hexameric tail terminator protein, gp14, and 26 stacked hexamer rings of TTP gp15 (Fig. 1B; Fig. S1C). The tail terminator gp14 terminates the growth of the tail tube and is connected to the hexameric stopper, exhibiting topological identity to that observed in siphophages lambda and T5 (36, 37). All TTP rings form a right-handed helix with an axial rise of approximately 38.5 Å and a twist of approximately 14.8° (Fig. 2A), forming a super-flexible structure that facilitates efficient screening of host receptors for siphophages. The channel within the tail tube is occupied by trimeric TMP gp19, and the distal tail is connected to the tail tip (Fig. 2C).

The Avs-1 tail tip, located at the distal end of the tail, is essential for host recognition, cell adsorption, and DNA ejection (39). The tip contains four proteins: the hexameric DTP gp20, the threefold hub protein gp23, the threefold insertion protein gp21, and the trimeric spike protein gp43 (Fig. S1). The DTP ring gp20 exhibits a TTP-like fold analogous to that of TTP gp26 in Avs-1, with the side connected to three trimeric spikes (Fig. S1). The hub gp23 is coupled with the insertion protein gp21, forming an inverted cone-like structure that seals the distal end of the tail (Fig. S1).

### Genome analysis of Avs-1

**Fig 3:**
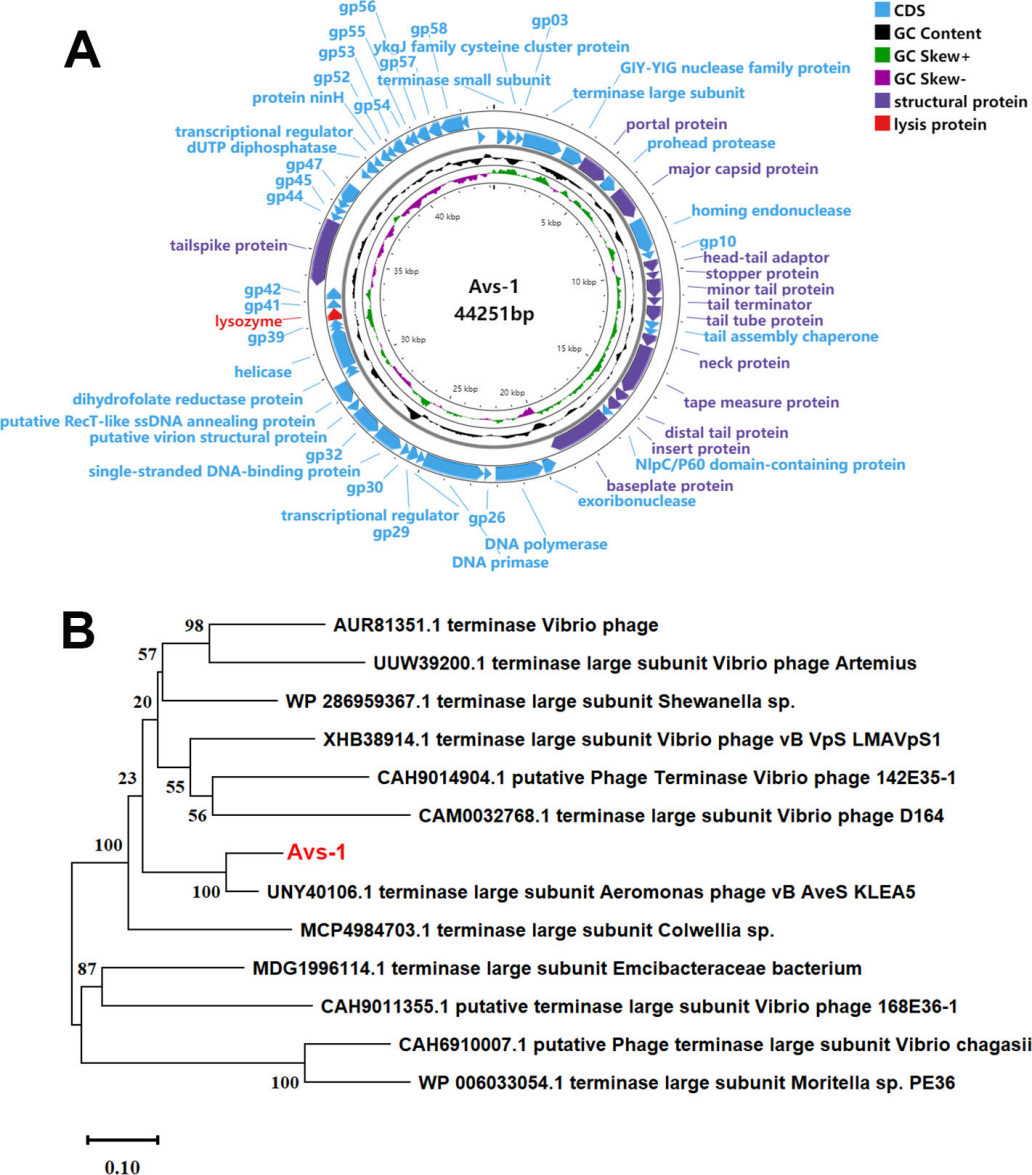
Genome analysis of the phage Avs-1. (**A**) Graphical representation of the phage ASG01. Circles show (from the outside to the inside) (1) the coding seqs counter−clockwise; (2) the coding seqs clockwise; (3) G + C% content; and (4) GC skew. Values greater than zero are in green, while those lower than zero are in purple; (5) physical map scaled in kb. The red font and purple font in the outermost ring represent the encoded cleavage and structural proteins, respectively. (**B**) Neighbor-joining phylogenetic tree based on the terminase large subunit amino acid sequence of phage Avs-1.

The whole genome of phage Avs-1 consisted of a circular double-stranded DNA, with a full length of 44,364 bp and a GC content of 48.17% (Fig. 3A). The genome encodes 60 putative ORFs, of which 26 proteins have assigned functions, and can be categorized into four functional modules: structure/assembly module, replication module, lysis module, and transcriptional regulation module. The structure/assembly module contains the small and large terminase subunit, portal protein, prohead protease, major capsid, neck protein, and tail proteins (Fig. 3A), which is consistent with the results of the overall structural analysis of the bacteriophage (Fig. 2D through F). Genes in the replication module include DNA polymerase and primase, endonuclease, and exodeoxyribonuclease (Fig. 3A). In the lysis module, *gp40* encodes a lysozyme designated as Lys40. However, no holin protein was predicted in the genome annotation results, likely due to its small molecular weight and variable structure, which may prevent existing annotation software from correctly identifying it (40). The transcriptional regulation module encodes two annotated transcription regulatory proteins (Fig. 3A). Phage Avs-1 does not encode any lysogenic phage-related proteins (Fig. 3A), such as transposases or integrase, suggesting that it is a strictly lytic phage (41). In addition to its crucial role in phage DNA packaging, the terminase large subunit was also one of the most conserved phage genes which was often used to describe the evolutionary relationship between phages (42). Phylogenetic analysis based on the large subunit of terminase revealed that Avs-1 shares the closest genetic relationship with *Aeromonas* phage vB_AveS_KLEA5 (Fig. 3B), exhibiting 88.25% sequence similarity. Interestingly, whole-genome sequence alignment via BLAST showed these two phages also share the highest homology, with a sequence similarity of only 62.66%, indicating that Avs-1 is a novel species of phage (43).

### Mutation of the *manB* gene causes resistance to Avs-1

**Fig 4:**
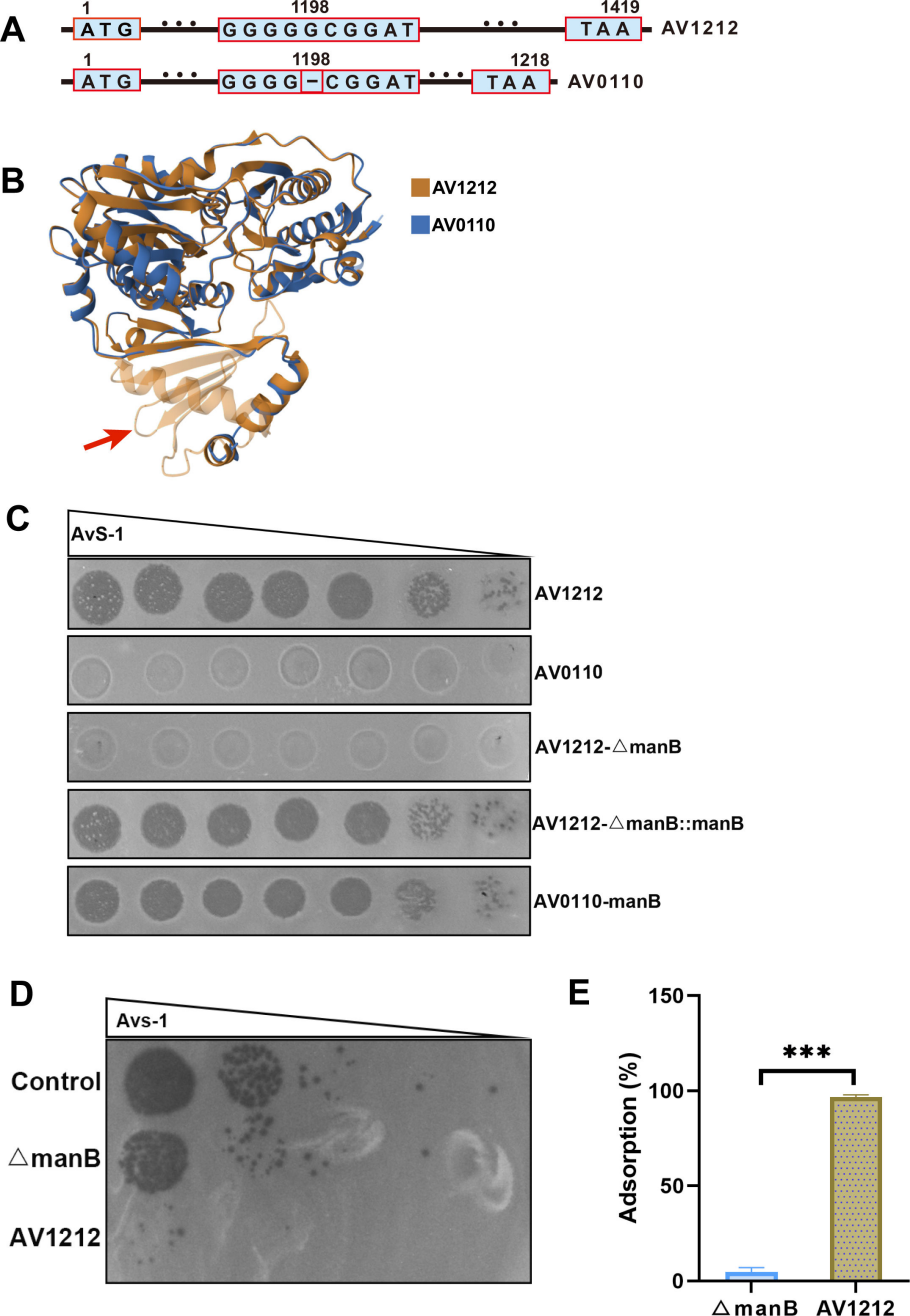
Identification and functional verification of the mutant gene manB in AV0110. (**A**) Comparison of the sequence of *manB* gene in AV1212 and AV0110 genome. (**B**) Comparison of the tertiary structure of ManB protein in AV1212 and AV0110. The red arrow in the lower left corner shows that ManB protein in AV0110 lacks the C-terminal domain due to premature termination of translation caused by mutation. (**C**) Plaque assay verified the sensitivity of bacteria to phage Avs-1. Analysis of plaque (**D**) and adsorption rate (**E**) of Avs-1 on the adsorption capacity of AV1212 and AV1212-△manB. The data were analyzed using two-way ANOVA. *** indicates *P* < 0.001.

During an experiment, the co-culture solution of phage and bacteria exhibited turbidity once again following clarification. Subsequently, the 16S ribosomal RNA (16S rRNA) gene sequence was determined for the purified colony, revealing its identification as a strain of *A. veronii* (designated AV0110). Notably, AV0110 demonstrated insensitivity to phage Avs-1 based on double-layer plate testing. Genome sequence analysis of AV1212 and AV0110, as well as the secondary sequencing verification of the mutation region, revealed mutations in two genes in the genome AV0110, namely, (i) a synonymous mutation in the *HscA* gene (position 1873064 to 1874911 in AV1212 genome) encoding the Fe-S protein assembly chaperone (Fig. S2) and (ii) a frameshift mutation in the gene *manB* (position 3202231 to 3203649 in AV1212 genome) encoding phosphomannomutase (PMM) (Fig. 4A), which causes premature termination of translation and leaves the protein lacking the C-terminal domain (Fig. 4B). The product of the *manB* gene, PMM, has been reported to be involved in the synthesis of O-antigen in lipopolysaccharide (LPS) (44), which was a common phage adsorption receptor (45, 46). Therefore, the mutation of gene *manB* may be responsible for the resistance of *A. veronii* AV0110 to phage Avs-1.

To verify whether the *manB* mutation in AV0110 conferred phage resistance, we constructed a *manB* deletion mutant of AV1212 (AV1212-△manB) and cloned the *manB* gene and its native promoter into vector pBBR1 for complementation in AV1212-*△manB* (AV1212-△manB::manB) and expression in AV0110 (AV0110-manB). Plaque assay results suggested that the deletion of *manB* resulted in phage resistance of AV1212, and the complementation of the *manB* restored the sensitivity to phage (Fig. 4C). Furthermore, AV0110 restored sensitivity to Avs-1 after expressing the *manB* gene. (Fig. 4C). To identify the specific stage of the phage life cycle at which resistance occurs, we performed an adsorption assay. The results revealed that, compared to the WT strain, AV1212-△manB completely prevented phage adsorption (Fig. 4D), with an adsorption rate close to zero (Fig. 4E). These results revealed that phage resistance in AV0110 was caused by a frameshift mutation in the *manB,* which led to changes in phage receptor structure, preventing phage adsorption.

### Antibacterial activity of phage Avs-1 *in vivo* and *in vitro*

**Fig 5:**
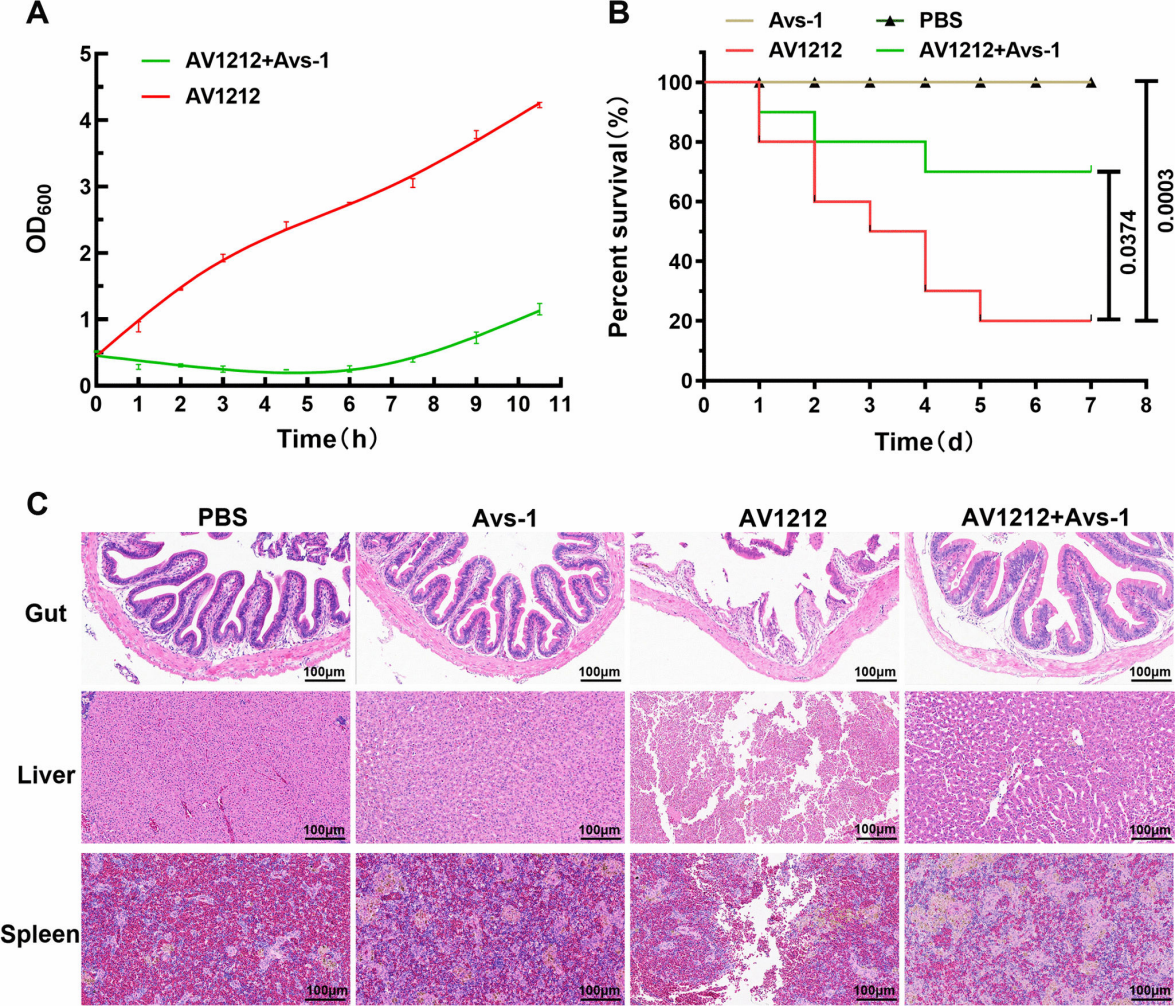
*In vitro* antimicrobial activity of bacteriophage Avs-1 and its protective effect on tilapia infected with *A. veronii*. (**A**) *In vitro* antimicrobial curve of bacteriophage Avs-1. Growth was monitored by measuring OD_600_; standard deviation to the mean is shown for each timepoint. (**B**) The influence of phage on the survival rate of tilapia infected with *A. veronii*. (**C**) Histological findings of the gut, liver, and spleen of tilapia. The experiment consists of four groups in total, with 10 fish per treatment group, and each treatment group has three replicates. Survival curves were analyzed using the log-rank test, * indicates *P* < 0.05; *** indicates *P* < 0.001.

To evaluate the application value of Avs-1 for prevention and control of bacterial diseases, we firstly assessed its *in vitro* antibacterial activity. The results showed that the growth of *A. veronii* AV1212 was continuously inhibited within 7.5 h after the addition of phage Avs-1 (Fig. 5A). Subsequently, to further investigate the effectiveness of Avs-1 as an antibacterial agent within the field of aquaculture, we studied its protective effect on tilapia infected with *A. veronii* AV1212. Our findings revealed that tilapia maintained a 100% survival rate in the control group, which had been injected with either phosphate buffer solution (PBS) or Avs-1 (Fig. 5B). However, tilapia treated with *A. veronii* AV1212 alone began to die from the next day, accompanied by abdominal swelling and bleeding, which led to the final survival rate of only 20%. In contrast, when the tilapia were injected with both *A. veronii* AV1212 and the phage Avs-1, the bleeding symptoms were significantly relieved, and the survival of tilapia remained stable on the fourth day. Ultimately, a commendable 70% of the tilapia survived, which was 40% higher than the survival rate of the group that had only been injected with *A. veronii* AV1212 (Fig. 5B). Histopathological examination results revealed that intestinal, splenic, and hepatic tissues exhibited no pathological symptoms following injection of PBS or Avs-1, with intact structures and tightly arranged cells (Fig. 5C). In contrast, significant lesions were observed in all tissues following injection of AV1212 alone: gut villi and mucosa underwent sloughing; hepatocytes showed extensive necrosis and disorganized arrangement, accompanied by eosinophilia and inflammatory cell infiltration; and the spleen displayed disrupted tissue architecture and cellular vacuolization (Fig. 5C). Following treatment with Avs-1, all pathological changes were significantly alleviated, and the overall tissue architecture was largely restored to normal—although partial sloughing of the intestinal mucosa persisted (Fig. 5C). Notably, splenocytes and hepatocytes maintained intact structures (Fig. 5C). These results demonstrate that Avs-1 is a safe agent for controlling bacterial diseases, as it can effectively mitigate the pathological damage induced by *A. veronii* in tilapia.

### Lytic activity preliminary analysis of Lys40 in *E. coli*

**Fig 6:**
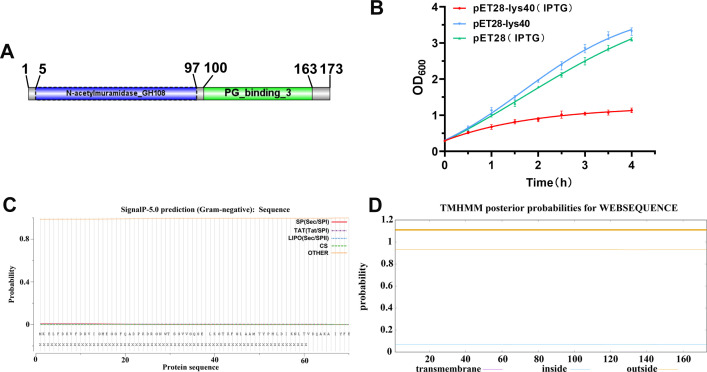
Bioinformatic and lytic activity analysis of Lys40. (**A**) Lys40 protein domain prediction. (**B**) Effect of Lys40 expression on the growth of *E. coli*. Growth was monitored by measuring OD_600_ every 30 min for 4 h; standard deviation to the mean is shown for each time point. (**C**) Signal peptide prediction of Lys40. (**D**) Transmembrane structure prediction of Lys40.

Within the genome of Avs-1, the gene *gp40* was predicted to encode a lysozyme (Lys40). Lys40, consisting of 173 amino acids, contains an N-acetylmuramidase domain (5–97 aa) at N-terminus, which hydrolyzes the glycan bond in peptidoglycan of bacterial cell walls, and a peptidoglycan (PG)‐binding domain (100–163 aa) at the C-terminus for binding to bacterial receptors (Fig. 6A). To assess the lytic activity against *E. coli*, the gene *lys40* was cloned into pET28a and heterologously expressed in *E. coli* BL21(DE3). After induction by IPTG, Lys40 could significantly inhibit the growth of *E. coli* but could not lyse it (Fig. 6B). Moreover, IPTG had a slight inhibitory effect on *E. coli* cells (Fig. 6B). Signal P5.0 and TMHMM analyses indicated that Lys40 does not contain signal peptides (Fig. 6C) nor transmembrane domains (Fig. 6D), suggesting that Lys40 may cross the bacterial inner membrane into the periplasmic space via other proteins.

## DISCUSSION

In recent years, a rapid development of aquaculture has made farmed fish one of the most significant affordable sources of protein foods worldwide (47). *A. veronii*, a significant fish pathogen, has caused serious losses in aquaculture. Bacteriophages and phage-derived antibacterial proteins have been recognized as promising alternatives to antibiotics for the application of infections caused by multidrug-resistant bacteria such as *A. veronii* (48–50). In this study, a novel lytic phage Avs-1 was isolated and showed potent virulence in *A. veronii*. Avs-1, which contains an icosahedral head and a long non-contractile tail, is a typical phage of the *Siphoviridae* family. Its short incubation period and long lysis period are conducive to the rapid prevention and control of *A. veronii*. The study of the Avs-1 genome and structure provides novel insights into its infection mechanism and opens new avenues for the biological control of bacterial diseases. At present, there are relatively few reports on *A. veronii* phage compared with other *Aeromonas* spp. The isolation of the novel *A. veronii* phage Avs-1 is of great significance for future phage therapy of *A. veronii* infections in aquaculture.

The differential gene *manB* was identified by genomic analysis of wild-type (WT) *A. veronii* AV1212 and phage resistance mutant *A. veronii* AV0110, and we proved that the *manB* mutation confers phage resistance by preventing phage adsorption. ManB is a phosphomannomutase (PMM) that has been shown to be vital for the synthesis of the common phage receptor O-antigen (51). Mutations in genes encoding phage receptors or enzymes related to receptor synthesis, leading to the inability of phages to adsorb, are the most common bacterial resistance strategy during phage–host interaction (52). However, such mutations in bacteria reduce their viability, such as reduced virulence or loss of antibiotic resistance. For example*, Acinetobacter baumannii* acquired phage resistance through capsular deletion caused by mutations in genes encoding glycosyltransferase 29 and glucose-6-phosphate isomerase, but at the same time, it restores the resistance to β-lactam antibiotic (53). Phage modification, on the other hand, might be a promising way to suppress phage resistance. Phage tail fibers (54), usually acting as receptor-binding proteins (RBPs) that bind to bacterial receptors, could be modified by mutagenesis or gene editing to suppress bacterial resistance to phage infection or to expand host range (55, 56). Furthermore, the high-resolution structural characterization of Avs-1, particularly of its tail spike protein, provides an atomic-level blueprint for understanding host recognition—a valuable asset for future receptor-binding protein (RBP) modification to counter bacterial resistance.

We demonstrated that the potent, sustained bactericidal activity of Avs-1 against AV1212 *in vitro*, which achieved complete eradication within 7.5 h, translates into a significant protective effect *in vivo* (70%). Although the observed protection is not absolute, it demonstrates that Avs-1 can serve as a promising antimicrobial agent. These findings provide a cornerstone for future development, and optimizing administration regimens, such as dosage and timing, will be necessary to fully realize its clinical potential. Meanwhile, leveraging the genome and structural characteristics of Avs-1, genetic engineering modifications are performed to either infect drug-resistant strains or enhance bactericidal effects, thereby improving therapeutic efficacy (57, 58).

In this study, the expression of *lys40* alone could not lyse *E. coli* intracellularly, suggesting that bacteriophage Avs-1 genome should contain holin-like genes which were not predicted. In addition, the modular structure of Lys40 is conducive to the development of various chimeric endolysins. For example, fusing with a short peptide with membrane-penetrating capabilities in the N-terminal of endolysin enables it to penetrate the outer membrane of Gram-negative bacteria, which usually prevents the penetration and degradation of endolysin (59). Moreover, the cell wall-binding domain (CBD) from endolysin fused with fluorescent protein has the ability of rapid detection of pathogens (60).

In summary, this study provides a comprehensive investigation of the Siphoviridae phage Avs-1, unveiling its genomic characteristics, unique Cryo-EM structure, the *manB*-mediated host resistance mechanism, and potential as an antibacterial agent. These findings not only deepen our understanding of phage-host interactions but also demonstrate that Avs-1 is a promising biocontrol agent to mitigate bacterial diseases caused by *A. veronii* in aquaculture, providing direction and tools for further therapeutic applications.

## MATERIALS AND METHODS

### Bacterial strains, plasmids, and culture conditions

*E. coli* DH5α*,* DH5α-λpir*,* BL21(DE3), and WM3064 were used for cloning, expression, and as the donor strains in conjugative transfer experiments. Plasmid pET28a and pBBR1 were used for recombinant endolysin expression and gene *manB*, respectively, and, pRE112 suicide vector was used to construct the gene knockout vector. Bacteria and phages were cultured in Luria–Bertani (LB) broth at 37°C with shaking, except for *A. veronii* AV1212 and AV0110 (30°C).

### Phage isolation and purification

Phage isolation was performed as previously described with some modifications (61). Briefly, sewage samples collected from a small creek at the foot of Yuelu Mountain in Changsha City, Hunan Province, were centrifuged at 4,000 rpm for 15 min, and the supernatant was filtered through a 0.22 μm filter. The filtrate was spotted onto the lawn of AV1212 bacteria. The above steps were repeated until phage plaques appeared. The double-layer agar plate method was used for the purification of phage (62). Briefly, plaques were picked and co-cultured with the host bacteria for 12 h at 30°C, then the cleared bacteria–phage co-culture was centrifuged at 9,000 revolutions per minute (rpm) for 15 min at 4°C. The supernatant is filtered through a 0.22 μm filter to remove bacteria. The top agar layer (0.7% agar) containing the filtrate mixed with host culture was poured onto the bottom agar (1.8% agar). Repeat the phage purification step until single-plaque morphology was observed. The host range was tested with the same method. Information regarding the tested strains is shown in Table 1.

### Determination of the multiplicity of infection (MOI)

The multiplicity of infection refers to the initial ratio of bacteriophages to host bacteria in an infection system. The multiplicity of infection was determined according to the method described by previously with some modifications (63). For each tested multiplicity of infection, equal volumes (1 mL each) of phage suspension and host bacterial culture were combined and then brought to a final volume of 100 mL with LB broth. After 4 h of incubation at 30°C, the mixture was centrifuged at 9,000 rpm and filtered through a 0.22 μm filter. The phage titer was determined by double-layer agar plate method, and the infection complex with the highest titer was selected as the best. All experiments were performed in triplicate.

### One-step growth curve

One-step growth curve assays were performed as described previously (64). Briefly, fresh *A. veronii* AV1212 culture (1.5 × 10^8^ CFU/mL) was mixed with phage at the MOI of 0.1 and allowed to adsorb for 15 min at 30°C. Then, the culture was centrifuged at 9,500 rpm for 10 min to remove unadsorbed phages, resuspended in 100 mL LB, and the dilution was incubated at 30°C. Samples were taken every 20 min and plated using the double-layer agar plate method to determine phage titer. The burst size was calculated as the ratio of the plateau phage titer to the initial bacterial count. Each assay was performed in triplicate.

### Transmission electron microscopy

The morphology of the phage was observed by transmission electron microscopy, as previously described with slight modifications (65). A purified phage suspension (~10¹⁰ PFU/mL) was loaded onto a carbon-coated copper grid, followed by negative staining with 2% phosphotungstic acid (Shhushi, Shanghai, China). The stained specimens were observed under a transmission electron microscope (Hitachi HT7700, Tokyo, Japan) at an acceleration voltage of 80 kV.

### Cryo-EM sample preparation and data collection

The sample preparation process is as described previously, with only minor modifications (36). A 3.5 µL aliquot of the purified phage Avs-1 was applied to graphene oxide (GO) grids. The grids were blotted using a Thermo Fisher Vitrobot under conditions of 100% humidity and 8°C for 3.5 s, then were plunge-frozen in liquid ethane and subsequently stored in liquid nitrogen. Data were acquired on a Thermo Scientific Krios G3i transmission electron microscope equipped with a Gatan K3 direct electron detector. A total of 3,068 micrographs were collected at a magnification of 40,000×, which corresponds to a calibrated pixel size of 1.248 Å. Each movie stack was fractionated into 32 frames with a total electron exposure of approximately 32 e−/Å².

### Image processing and 3D reconstruction

All image processing and 3D reconstruction were performed using cryoSPARC (66). The workflow commenced with contrast transfer function (CTF) correction applied to 3,068 motion-corrected micrographs. From 2,363 micrographs, Avs-1 head particles were initially picked using the “Blob Picker.” Following 2D classification to select particles, these were used as templates for automated capsid particle picking with the “Template Picker.” Subsequent 2D classification yielded 43,948 homogeneous particles (box size: 800 × 800 pixels). These particles underwent “Ab-Initio Reconstruction” with imposed icosahedral (I) symmetry, and the resulting initial model was refined through “Homogeneous Refinement” with I symmetry, yielding a final 2.98 Å resolution cryo-EM map of the DNA-filled capsid.

For the neck and tail tube structures, particles underwent symmetry expansion, volume alignment to a vertex, and duplicate removal. After particle extraction, 3D classification selected 35,569 particles containing the portal vertex. Subsequent “Homogeneous Refinement” under C6 symmetry produced the neck structure at a resolution of 3.35 Å. Subsequently, the map center was shifted to the tail tube region, and after another particle extraction, an initial model was refined under C3 symmetry, resulting in a 3.26 Å tail tube structure.

For the tip complex, template-based particle picking and 2D classification yielded 22,840 particles. An initial model was generated by “Ab-Initio Reconstruction” and iteratively refined using homogeneous, local, and local CTF refinement, achieving a 3.62 Å tip structure. These particles then underwent C6 symmetry expansion and center shifting to a spike protein. After duplicate removal and particle extraction, 3D classification identified two classes. Following alignment with the “Align 3D” tool and “Reconstruction” on C1, “Local Refinement” produced maps of the spike-DTP interface at 3.76 Å and the spike oscillation region at 3.58 Å resolution.

### Phage DNA extraction, sequencing, and bioinformatic analysis

Phage DNA was extracted using the phage DNA isolation kit (Abigen, Beijing, China), and genomes were sequenced by GENEWIZ (GENEWIZ, Suzhou, China). The whole genomes were sequenced with the Illumina (San Diego, CA, USA) HiScanSQ and assembled using SOAPdenovo v2.01 (67). The tool ORF (open reading frame) Finder (https://www.ncbi.nlm.nih.gov/orffinder/) was used to predict open reading frames (ORFs), and a Uniprot (https://www.uniprot.org/blast) analysis was performed to assign functional annotations to the predicted ORFs. The Proksee was used to create genome maps (68). The amino acid sequence of the Avs-1 large terminase subunit was analyzed using the BLAST tool (https://blast.ncbi.nlm.nih.gov/Blast.cgi). The 12 sequences exhibiting the highest homology were selected, and a phylogenetic tree of the phage large terminase subunit was constructed using MEGA11 software (69). Similarly, the BLAST tool was also used to analyze the sequence homology between the complete genome of phage Avs-1 and other phages.

### Isolation and identification of phage Avs-1-resistant bacteria AV0110

Single colonies were picked and cultured in LB broth. Using these bacteria as indicator strains, the double-layer plate method was used to detect whether they were resistant to phage Avs-1. The picked Avs-1-resistant bacteria were identified via colony polymerase chain reaction (PCR) and 16S ribosomal RNA gene fragment sequencing at Sangon (Sangon Biotech, Shanghai, China). The phage-resistant bacteria identified as *A. veronii* were continuously subcultured, and their phage resistance was detected by the double-layer plate agar method.

### *A. veronii* AV1212 and AV0110 genomes sequencing and bioinformatic analysis

*A. veronii* AV1212 and AV0110 genomic DNA were prepared using the Bacterial Genomic DNA Extraction Kit (Sangon Biotech, Shanghai, China). The whole genomes of *A. veronii* AV1212 and AV0110 were sequenced and assembled using Oxford Nanopore GridION. The whole genomes of the AV1212 and AV0110 were compared and analyzed based on reads and single nucleotide polymorphism (SNP) to analyze the mutation types of the mutated genes by Nextomics Biosciences Co., Ltd. (Nextomics Biosciences, Wuhan, China). CRISPR-Cas structures were predicted using MinCED (https://github.com/ctSkennerton/minced), and the PHAST web server (https://phastest.ca/) was used to predict prophage sequences. Protein 3D structure model prediction was generated using SWISS-MODEL (https://swissmodel.expasy.org/), and pairwise structure alignment was used to align protein 3D structures (https://www.rcsb.org/alignment).

### Construction of engineered strains

*A. veronii* AV1212 gene *manB* deletion mutant was constructed by allelic exchange using the suicide vector pRE112. Briefly, the upstream (primers: *up*F and *up*R) and downstream (primers: *dn*F and *dn*R) homologous arm fragments of gene *manB* and Cm resistance gene (primers: *cm*F and *cm*R) were amplified, and the pRE112 vector was digested with the restriction enzymes *Kpn* I (New England Biolabs, Ipswich, MA, USA) and *Sac* I (New England Biolabs, Ipswich, MA, USA). These four fragments were ligated using Vazyme One-Step Cloning Kit (Vazyme, Nanjing, China) and then transformed into DH5α-λpir to construct the knockout vector pRE112-△manB. This vector was first introduced into *E. coli* WM3064 and then conjugated into *A. veronii* AV1212. Positive deletion mutants were selected on LB agar plates containing chloramphenicol (30 µg/mL) and 10% (wt/vol) sucrose and confirmed using PCR and DNA sequencing.

For complementation and expression, the *manB* gene and its native promoter region (primers: *manB*F and *manB*R) were amplified and cloned into the plasmid pBBR1-MCS2 via *Xho* I (New England Biolabs, Ipswich, MA, USA) and *Hind* III (New England Biolabs, Ipswich, MA, USA) to construct vector pBBR1-MCS2-manB. Similarly, the vector was introduced into *E. coli* WM3064 and then conjugated into deletion mutant strain *A. veronii* AV1212-△manB and the phage-resistant strain AV0110 for gene complementation and expression, respectively.

Putative endolysin gene in phage Lys40 was cloned into the vector pET28a via *Hind* III (New England Biolabs, Ipswich, MA, USA) and *EcoR* I (New England Biolabs, Ipswich, MA, USA) and introduced into *E. coli* BL21, which allowed the expression of *lys40* to be induced under the control of the lac promoter using IPTG as the inducer. The primer sequences used are shown in Table S1.

### Sensitivity analysis of strains to phage

The plaque assay was used to detect the phage sensitivity. The five tested *A. veronii* strains (AV1212, AV1212-△manB, AV1212-△manB::manB, AV0110, and AV0110-manB) were cultured overnight at 37°C and 250 rpm. To perform plaque assays, 1 mL overnight cultures were decanted onto an LB agar plate and left to dry for 15 min under microbiological flame. The phage suspensions were 10-fold serially diluted with PBS, and 3 μL of each dilution was used to spot on the bacterial lawn to evaluate sensitivity of strains to phage. Each assay was performed in triplicate.

### Bacteriophage adsorption assay

The bacteriophage adsorption assay was performed as previously described with some modifications (70). Briefly, bacterial cultures (AV1212 and △manB) grown overnight were resuspended with fresh LB broth and adjusted to OD_600_ = 0.6. Then, Avs-1 was added to the bacterial suspension at an MOI of 0.1, and the mixture was incubated at 30°C for 20 min to ensure sufficient adsorption. The mixture was centrifuged at 12,000 rpm for 10 min, and the supernatant was immediately filtered through a 0.22 μm pore diameter membrane filter to remove residual lysates. Five microliters of the 10-fold serially diluted supernatant were spotted onto an AV1212 bacterial lawn, and the phage titer was calculated. The Avs-1 filtrate without bacterial culture was used as the control group. The percentage of adsorption was calculated as [(control group plaques − the plaques after adsorption) / control group plaques] × 100%. The final adsorption rate was obtained from three independent experiments.

### Determination of the *in vitro* antibacterial effect of phage Avs-1

Fresh *A. veronii* AV1212 culture (4 × 10^9^ CFU/mL) was added into 100 mL of LB medium in a 1% ratio. After 2 h of cultivation (OD_600_≈0.5), 1 mL of the phage filtrate (1 × 10^8^ PFU/mL) was added, and the mixture was incubated at 30°C with constant shaking at 180 rpm. The OD_600_ of the culture medium was determined at different times. The bacterial culture without added bacteriophage served as a control group. Each assay was performed in triplicate.

### Protective effect of phage Avs-1 on the tilapia infected with *A. veronii*

Healthy tilapia were obtained from a commercial fish farm (Guangxi, China). A total of 120 healthy, uniformly sized tilapia weighing approximately 20 g were randomly assigned to four groups, each with three replicates. In all experimental groups, the test preparations were administered via intraperitoneal injection at a uniform dosage of 100 μL (5 μL/g). The concentration of AV1212 was approximately 1 × 10^9^ CFU/mL, and the phage preparation concentration was 1.5 × 10^8^ PFU/mL. The PBS control group received only PBS injection to avoid interference from accidental tilapia mortality caused by experimental procedures. The phage control group received only phage injection to verify the safety of the phage preparation. In the pathogen AV1212 challenge control group, bacteria were injected first, followed by PBS injection after 3 h to ensure consistency in experimental procedures. Finally, in the phage treatment group, bacteria were injected first to simulate natural infection, followed by phage injection after 3 h. The therapeutic effect of Avs-1 was analyzed through the survival rate and histopathological damage of tilapia. Statistical significance was assessed using the log-rank (Mantel-Cox) test. Three days after the initial injection, one tilapia was randomly dissected from each group to harvest kidney, liver, and intestinal tissue samples. The collected tissues were fixed in 4% paraformaldehyde and submitted to Wuhan Sevier Biotechnology Co., Ltd. for histopathological examination, including tissue embedding, sectioning, and hematoxylin and eosin (H&E) staining.

### Bioinformatics analysis of endolysin Lys40

The domain structure of Lys40 was annotated using the Interpro (https://www.ebi.ac.uk/interpro/). TMHMM 2.0 (http://www.cbs.dtu.dk/services/TMHMM/) was used to predict the transmembrane protein structure domain. Signal P5.0 (http://www.cbs.dtu.dk/services/SignalP/) was used to analyze its signal peptide.

### Activity assays of Lys40 in *E. coli*

A 200 μL *E. coli* BL21 (pET28a-lys40) overnight cultures was added to 20 mL LB broth and cultured 2 h at 37°C and 250 rpm and then induced with isopropyl β-D-1-thiogalactopyranoside (IPTG; Coolaber, Beijing, China) at a concentration of 0.5 mM at 37°C for 4 h. The BL21 (pET28a-lys40) strain without IPTG and the BL21 (pET28a) strain with IPTG alone were used as two control groups. The optical density at 600 nm (OD_600_) of the culture was detected every 30 min, and the experiment was repeated three times.

### Statistical analysis

Statistical analysis was performed using GraphPad Prism v8.0.2 (GraphPad Software, USA). Data were evaluated using two-way ANOVA or the log-rank test for survival curves, unless otherwise indicated. ns (no significance) indicates *P* > 0.05; * indicates *P* < 0.05; ** indicates *P* < 0.01; *** indicates *P* < 0.001; and **** indicates *P* <0.0001.

## Data Availability

The complete genome sequences of AV1212 and AV0110 have been submitted to GenBank under accession numbers CP196963 and CP196964. The complete genome sequence of phage Avs-1 has been uploaded to GenBank under accession number PX024133. The complete genomes of AV1212, AV0110, and Avs-1 raw sequence reads have been submitted to the Sequence Read Archive (SRA) under accession numbers SRR37013324 (AV1212), SRR37011204 (AV0110), and SRR37018936 (Avs-1). Cryo-EM maps have been deposited at the Electron Microscopy Data Bank under accessions EMD-67163, EMD-67164, EMD-67165, EMD-67166, EMD-67167, and EMD-67168. The source data for this paper are available on Figshare (10.6084/m9.figshare.30662273).

## SUPPLEMENTAL MATERIAL

Supplemental material aem.02334-25-s0001.docx Fig. S1 and S2; Table S1.

ASM does not own the copyrights to Supplemental Material that may be linked to, or accessed through, an article. The authors have granted ASM a non-exclusive, world-wide license to publish the Supplemental Material files. Please contact the corresponding author directly for reuse.
