# Supplementary material for: Isolation and characterization of Avs-1, a bacteriophage effective against the aquaculture pathogen Aeromonas veronii
Source: Appl Environ Microbiol. 2026 May 13;92(6):e02334-25. doi: 10.1128/aem.02334-25 (PMC13274417; doi:10.1128/aem.02334-25)
Supplement: Supplemental material — Fig. S1 and S2; Table S1. [file aem.02334-25-s0001.docx]

## Fig S1. Density maps of the 10 proteins of Avs-1.

(A) Cut-open view of the asymmetric structure of Avs-1. (B) Density maps of dodecameric portal, dodecameric adaptor, and hexameric stopper. (C) Density maps of hexameric tail terminator protein (TTP) and 5 stacked hexamer rings of TTP. (D) Density maps of tail tube and tail spike. The side of the tail tube protein is connected to three trimeric spikes. (E) Density maps of hexameric distal tail protein, threefold hub protein, threefold insertion protein, and trimeric spike protein. (F) Density maps of the distal tail, hub, insertion protein. Hub protein and insertion protein form an inverted cone-like structure.

## Fig S2 Fig. Comparison of the sequence of *manB* gene in AV1212 and AV0110 genome.

The mutation occured at base 417 and was a synonymous mutation.

## Table S1. Primers used in this study.

| **Primers** | **Primer sequences (5'-3')** |
| --- | --- |
| *up*F | CAAGCTTCTTCTAGAGGTACCTAGAGGTCGACATCGATCAC |
| *up*R | CAGCCCTTGCGCCCTGAGTGCCCTATTCAGGTGTAAAAGGA |
| *cm*F | GCACTCAGGGCGCAAGGGCTG |
| *cm*R | CATAGAATATCTAGCTAGAGTTACGCCCCGCCCTGCCACTC |
| *dn*F | CTCTAGCTAGATATTCTATG |
| *dn*R | CATGAATTCCCGGGAGAGCTCTGCTATGAACGGTAAGCTAG |
| *manB*F | GTACAAGCTTATCGTGGAAAAGAGGTTCAT |
| *manB*R | CATGCTCGAGTCATAACAAAAAGCGCACAG |
| *lys40*F | GGAATTCATGAAAGAACTGTTTGACAAGGT |
| *lys40*R | CCCAAGCTTTCAGTTATCCTGTGCAGCAT |





**S1 Fig. Density maps of the 10 proteins of Avs-1.** (A) Cut-open view of the asymmetric structure of Avs-1. (B) Density maps of dodecameric portal, dodecameric adaptor, and hexameric stopper. (C) Density maps of hexameric tail terminator protein (TTP) and 5 stacked hexamer rings of TTP. (D) Density maps of tail tube and tail spike. The side of the tail tube protein is connected to three trimeric spikes. (E) Density maps of hexameric distal tail protein, threefold hub protein, threefold insertion protein, and trimeric spike protein. (F) Density maps of the distal tail, hub, insertion protein. Hub protein and insertion protein form an inverted cone-like structure.





**S2 Fig. Comparison of the sequence of *manB* gene in AV1212 and AV0110 genome.** The mutation occured at base 417 and was a synonymous mutation.
